# Supplementary figures and images for: Integrated Framework for Probing Multimodal Protein Foundation Models with Structure-Functional Interpretability Analysis in Detection of Allosteric Binding Sites
Source: bioRxiv. 2026 Jul 7:2026.07.02.736203. Preprint. [Version 1] doi: 10.64898/2026.07.02.736203 (PMC13370958; doi:10.64898/2026.07.02.736203)

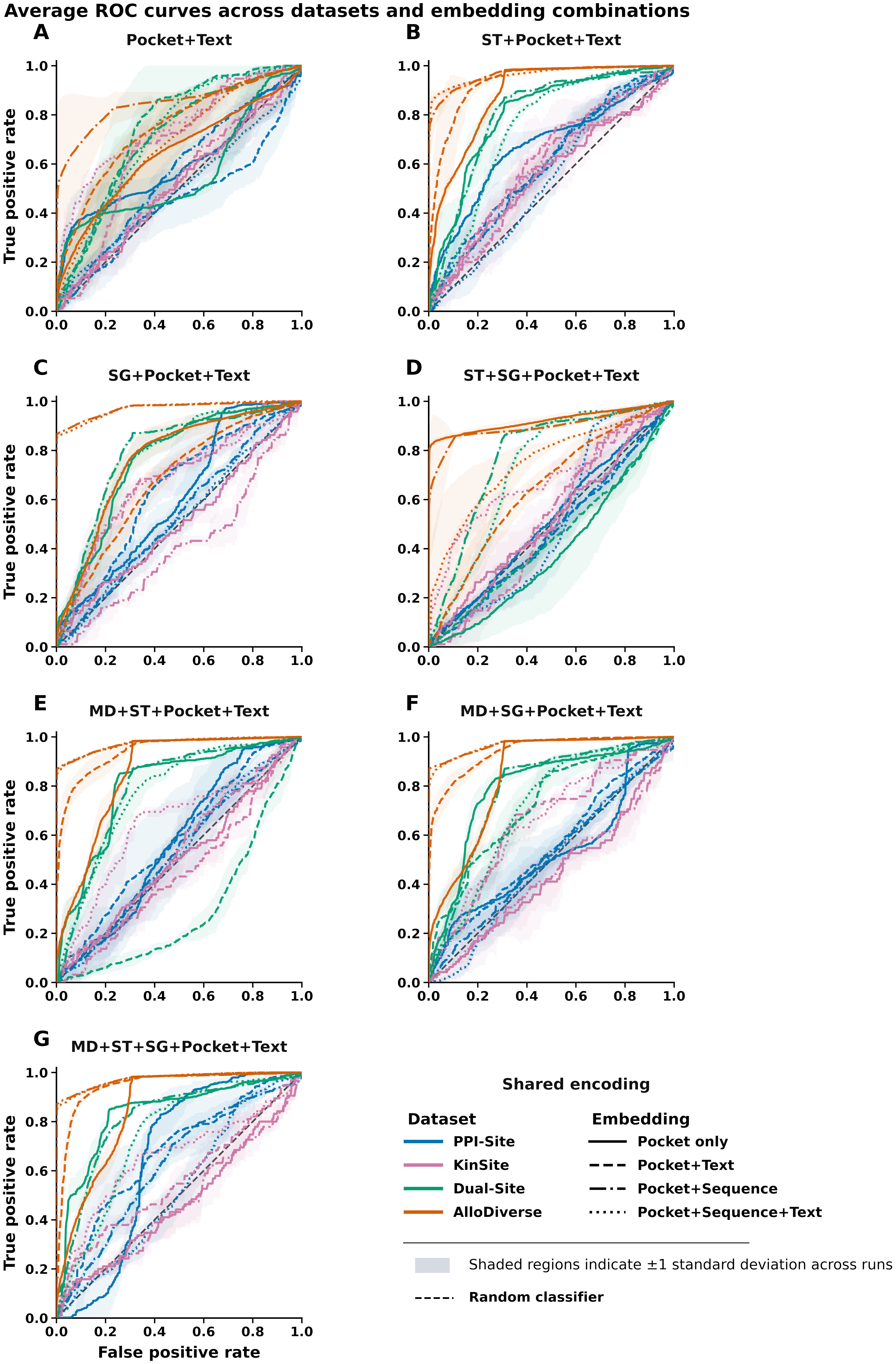

Supplement: Supplement 1 [file media-1.zip › SUPPORTING INFORMATION_BIORXIV/FigureS1_JCIM_SUBMISSION.tif]

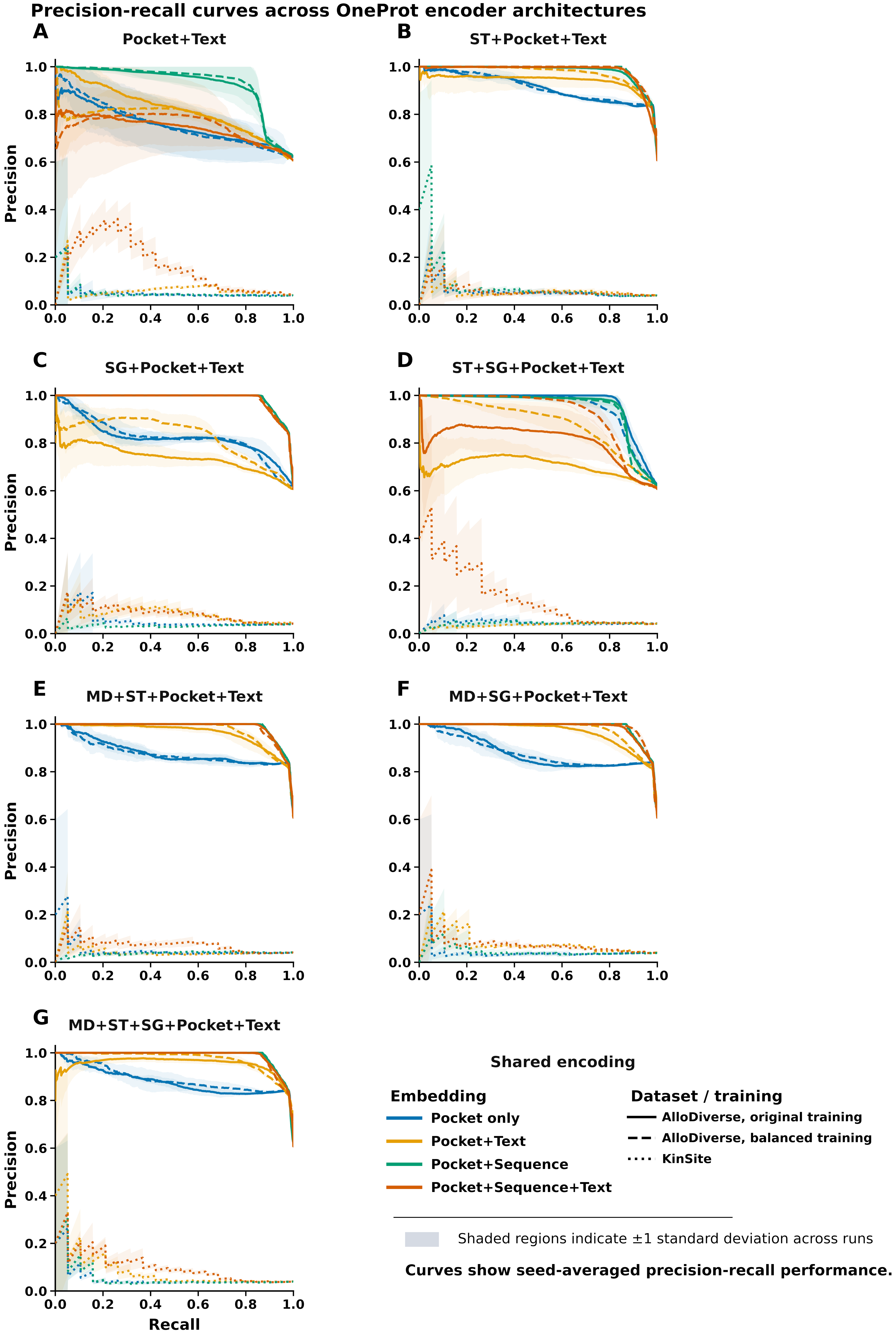

Supplement: Supplement 1 [file media-1.zip › SUPPORTING INFORMATION_BIORXIV/FigureS2_JCIM_SUBMISSION.tif]

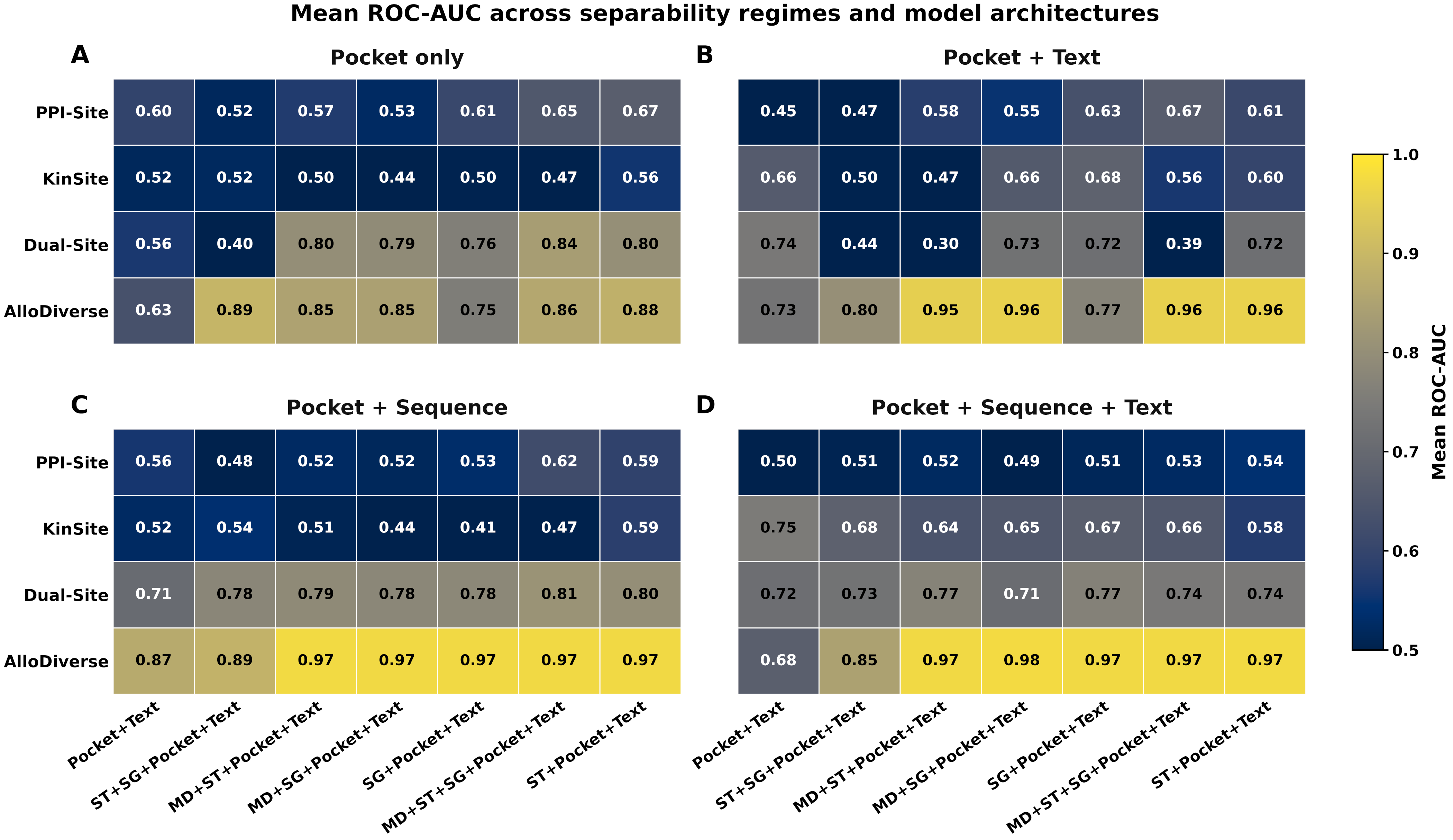

Supplement: Supplement 1 [file media-1.zip › SUPPORTING INFORMATION_BIORXIV/FigureS3_JCIM_SUBMISSION.tif]

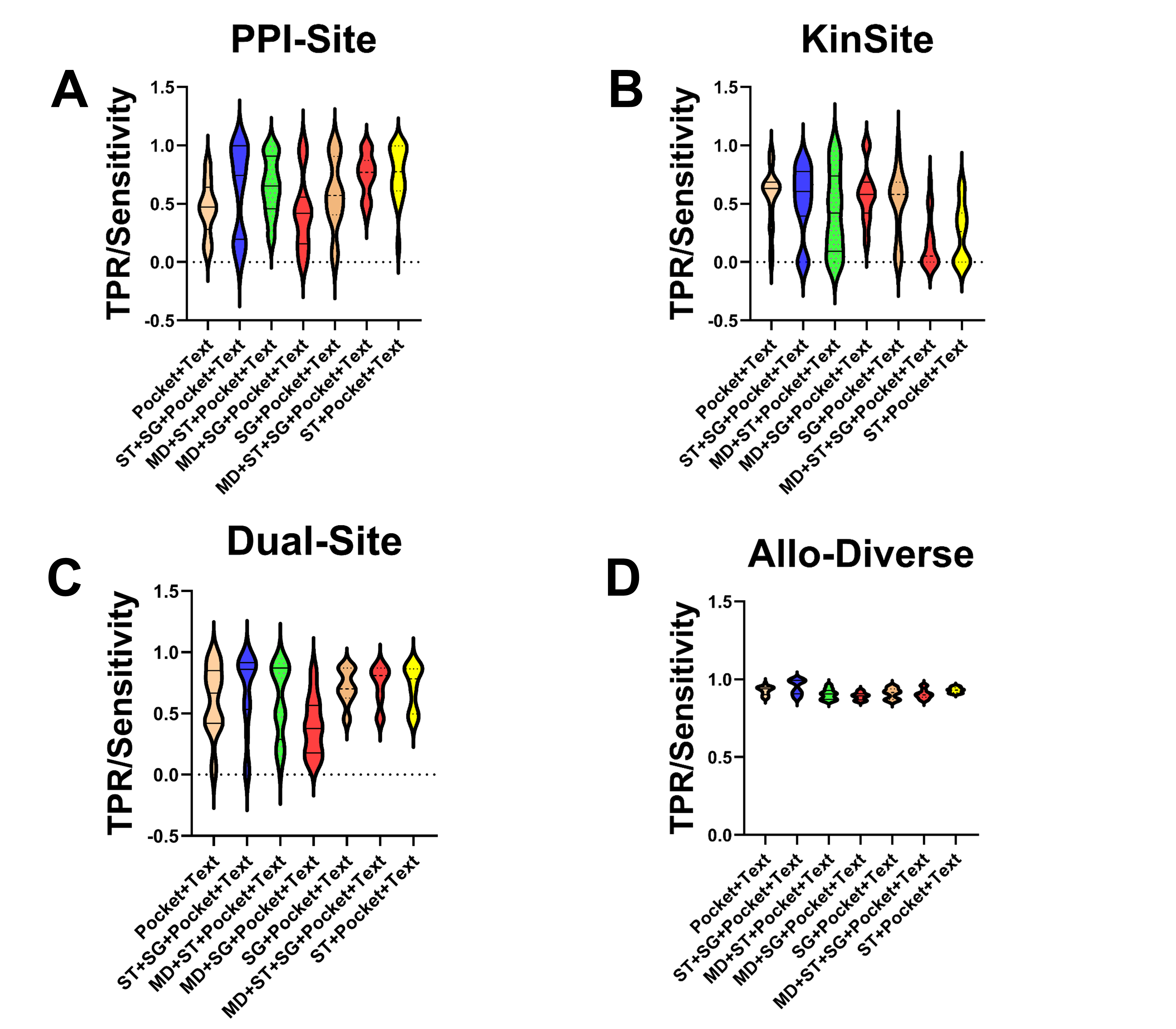

Supplement: Supplement 1 [file media-1.zip › SUPPORTING INFORMATION_BIORXIV/FigureS4_JCIM_SUBMISSION.tif]

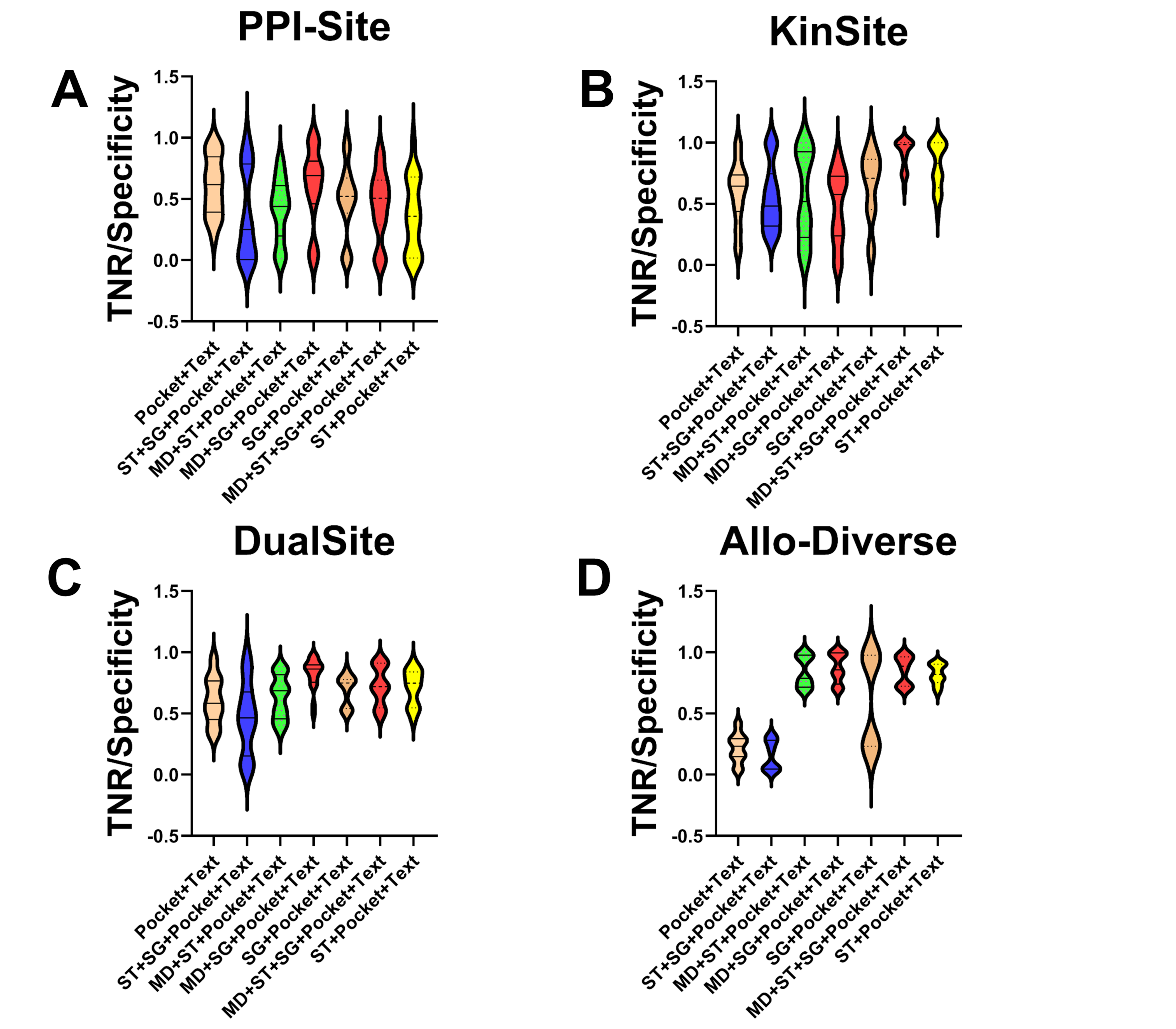

Supplement: Supplement 1 [file media-1.zip › SUPPORTING INFORMATION_BIORXIV/FigureS5_JCIM_SUBMISSION.tif]
